# Supplementary material for: Sex and strategy effects on brain activation during a 3D-navigation task
Source: Commun Biol. 2022 Mar 16;5:234. doi: 10.1038/s42003-022-03147-9 (PMC8927599; doi:10.1038/s42003-022-03147-9)
Supplement: Supplementary file 2 — Supplementary Information [file 42003_2022_3147_MOESM2_ESM.pdf]

Sex and strategy effects on brain activation during a 3D-navigation task

Isabel Noachtar[1*,#], Ti-Anni Harris[1#], Esmeralda Hidalgo-Lopez[1], Belinda Pletzer[1*]
#these authors contributed equally and should be considered shared first authors

[1]Department of Psychology and Centre for Cognitive Neuroscience
University of Salzburg, Hellbrunnerstr. 34, 5020 Salzburg, Austria

*corresponding authors:
Isabel Noachtar, isabel.noachtar@sbg.ac.at
Belinda Pletzer, belinda.pletzer@sbg.ac.at

Supplementary Information

Performance

The participant's overall performance during the navigation task improved over the three testing sessions with the last appointment showing the best performance ($b = 0.28$, $SE_b = 0.02$, $t_{(781)} = 18.04$, $p < 0.001$).

ROI-analyses

We observed a session effect in right hippocampus and bilateral caudate: the right hippocampus got less inhibited over the testing sessions meaning that during the first session it showed more inhibition and during the second and third session it was less inhibited ($b = 0.08$, $SE_b = 0.03$, $t_{(789)} = 3.20$, $p < 0.01$). The opposite direction of increasingly more inhibition over the testing session was found in the left caudate ($b = -0.09$, $SE_b = 0.03$, $t_{(789)} = -3.61$, $p < 0.001$) as well as in the right caudate ($b = -0.09$, $SE_b = 0.03$, $t_{(789)} = -3.25$, $p < 0.01$). There was no session effect in the retrosplenial cortex.

Whole-brain analyses

Activation analyses

Activation decreased with the number of sessions in a large cluster spanning the bilateral parietal cortices ([36, -40, 49], 1845 voxels, $T = 5.60$, $p_{FWE} < 0.001$). There were no increases in activation with the number of sessions.

Connectivity analyses
Connectivity to the left hippocampus and left caudate did not change significantly with the number of sessions.

Effects of session
Increased connectivity strength with the number of sessions was found from the right hippocampus to the right occipital pole ([27, -94, 10], 372 voxels, T = 5.80, p_{FWE} = 0.000). Connectivity strength from the right caudate to the right inferior occipital lobe ([33, -94, -2], 117 voxels, T = 5.11, p_{FWE} = 0.014), was decreased with the number of sessions.

Supplementary Table 1: Perspective effect in activation

Brain region	Side	MNI-coordinates (mm)			#voxels	*T*	*Peak level*
		X	Y	Z			p_{FWE}
**Allocentric > egocentric**							
Precuneus	L	-3	-61	46	9950	20.20	<0.001
**Egocentric > allocentric**							
Medial Frontal Cortex	R	0	44	-14	263	5.79	<0.001
Medial Precentral Gyrus	L	-12	-22	40	640	10.35	<0.001
Anterior Insula	L	-39	-4	13	1322	7.99	<0.001
Superior Parietal Lobule	R	24	-52	52	64	5.8	<0.001
Lingual Gyrus	L	-30	-49	-8	418	10.56	<0.001
Lingual Gyrus	R	30	-46	-8	2863	11.51	<0.001

Supplementary Table 2: Strategy effect in activation

Brain region	Side	MNI-coordinates (mm)			#voxels	*T*	*Peak level*
		X	Y	Z			p_{FWE}
**Euclidian > landmark**							
Middle frontal gyrus	L	-39	32	34	267	7.66	<0.001
Supplementary motor cortex	L	-3	-1	61	6729	19.95	<0.001
Precentral Gyrus	L	-27	-10	52	40	5.11	0.006
Middle cingulate gyrus	R	3	-25	43	99	6.42	<0.001
**Landmark > euclidian**							
Opercular part of Inferior Frontal Gyrus	R	39	23	22	202	10.39	<0.001
Middle Frontal Gyrus	L	-36	11	28	852	21.45	<0.001
Superior Temporal Gyrus	R	57	-7	-11	109	8.10	<0.001
Fusiform gyrus	L	-30	-40	-17	4511	23.20	<0.001
Anterior Insula	R	30	26	-2	49	4.93	0.013
Posterior Insula	L	-36	-10	13	44	5.11	0.006

Supplementary Table 3: Brain areas showing significant sex differences in BOLD-response during navigation

Brain region	Side	MNI-coordinates (mm)			#voxels	*T*	*Peak level*
		X	Y	Z			p_{FWE}

**Women > men**							
Middle Frontal Gyrus	R	27	8	49	128	8.17	<0.001
Precentral Gyrus	L	-30	-7	55	64	5.07	0.007
Precentral Gyrus	R	51	11	28	43	5.59	0.001
Postcentral Gyrus	L	-54	22	34	78	7.36	<0.001
Postcentral Gyrus	L	-30	-37	46	42	5.13	0.005
Superior Parietal Lobule	R	21	-49	64	66	6.66	<0.001
Superior Parietal Lobule	R	27	-64	37	70	5.54	0.001
Supramarginal Gyrus	R	54	-31	40	300	6.52	<0.001
Cuneus	R	3	-79	25	1119	8.68	<0.001
Middle Occipital Gyrus	L	-42	-79	19	232	7.17	<0.001
**Men > women**							
Superior Frontal Gyrus	L	-6	50	-2	701	6.93	<0.001
Superior Frontal Gyrus	L	-15	2	70	84	5.87	<0.001
Middle Frontal Gyrus	L	-36	44	22	224	6.37	<0.001
Middle Frontal Gyrus	L	-27	26	46	71	4.71	0.032
Supplementary Motor Cortex	L	-6	17	43	62	5.91	<0.001
Precentral Gyrus	L	-15	-37	46	111	7.4	<0.001
Precentral Gyrus	L	-45	-13	52	105	7.31	<0.001
Frontal Operculum	R	42	26	1	98	4.61	0.048
Rolandic Operculum	L	-54	2	10	115	7.09	<0.001

Supplementary Table 4: Perspective effect in LEFT hippocampus connectivity

Brain region	Side	MNI-coordinates (mm)			#voxels	*T*	*Peak level*
		**X**	**Y**	**Z**			p_{FWE}
**Allocentric > egocentric**							
Middle Cingulate Gyrus	R	3	8	34	122	5.29	0.005
Supramarginial Gyrus	R	60	-37	43	107	4.88	0.044
**Egocentric > allocentric**							
Precuneus	R	3	-61	28	306	6.09	<0.001

Supplementary Table 5: Perspective effect in RIGHT hippocampus connectivity

Brain region	Side	MNI-coordinates (mm)			#voxels	*T*	*Peak level*
		**X**	**Y**	**Z**			p_{FWE}
**Allocentric > egocentric**							
Middle Cingulate Gyrus	L	0	-1	37	129	5.90	<0.001
Middle Cingulate Gyrus	R	6	-22	43	216	5.13	0.012
Supramarginal Gyrus	R	60	-37	40	116	5.01	0.022
Fusiform Gyrus	L	-39	-46	-20	287	5.47	0.002
Anterior Insula	R	39	2	-2	207	5.45	0.002
Posterior Insula	L	-39	-13	-8	141	5.69	0.001
**Egocentric > allocentric**							

Middle Frontal Gyrus	R	30	23	52	89	5.23	0.007
Angular Gyrus	L	-39	-70	34	104	5.81	<0.001
Angular Gyrus	R	45	-61	25	121	5.83	<0.001
Precuneus	R	3	-61	25	370	7.84	<0.001

Supplementary Table 6: Perspective effect in LEFT caudate connectivity

**Brain region**	**Side**	**MNI-coordinates (mm)**			**#voxels**	***T***	***Peak level***
		**X**	**Y**	**Z**			p_{FWE}
**Allocentric > egocentric**							
Middle Frontal Gyrus	L	-42	2	49	737	7.23	<0.001
Supplementary Motor Cortex	L	-6	11	49	51	5.25	0.007
Superior Parietal Lobule	L	-33	-52	46	523	7.33	<0.001
Inferior Temporal Gyrus	L	-48	-58	-14	422	7.92	<0.001
Inferior Occipital Gyrus	R	27	-94	1	246	6.62	<0.001
Inferior Occipital Gyrus	L	-33	-91	-2	138	5.74	<0.001
Putamen	L	-18	8	1	376	9.42	<0.001
Putamen	R	18	11	-2	435	9.29	<0.001
**Egocentric > allocentric**							
Supplementary Motor Cortex	R	12	23	55	1590	6.04	<0.001
Supplementary Motor Cortex	L	-9	-16	49	203	5.05	0.018
Precentral Gyrus	R	27	-13	64	43	5.50	0.002
Parietal Operculum	R	60	-28	28	506	5.88	<0.001
Anterior Insula	L	-36	17	-14	212	7.19	<0.001
Anterior Insula	R	39	20	-14	570	6.10	<0.001

Supplementary Table 7: Perspective effect in RIGHT caudate connectivity

**Brain region**	**Side**	**MNI-coordinates (mm)**			**#voxels**	***T***	***Peak level***
		**X**	**Y**	**Z**			p_{FWE}
**Allocentric > egocentric**							
Middle Frontal Gyrus	L	-48	17	28	1394	8.10	<0.001
Superior Parietal Lobule	L	-33	-52	43	1118	8.29	<0.001
Inferior Temporal Gyrus	R	57	-52	-11	48	5.53	0.001
Middle Temporal Gyrus	L	-54	-55	-8	476	8.06	<0.001
Angular Gyrus	R	30	-61	46	95	4.90	0.039
Inferior Occipital Gyrus	L	-27	-94	-8	109	5.92	<0.001
Occipital Pole	R	27	-97	1	526	7.69	<0.001
Putamen	L	-18	8	1	492	9.46	<0.001
Putamen	R	18	8	1	404	8.21	<0.001
**Egocentric > allocentric**							
Orbital Part of the Inferior Frontal Gyrus	R	45	26	-11	1765	7.42	<0.001
Posterior Orbital Gyrus	L	-36	17	-17	582	7.01	<0.001
Postcentral Gyrus	L	-48	-19	34	276	5.22	0.008

Anterior Cingulate Gyrus	R	6	44	16	2000	6.94	<0.001
Middle Cingulate Gyrus	R	6	-22	43	458	6.27	<0.001
Lingual Gyrus	R	24	-46	-8	386	6.46	<0.001

Supplementary Table 8: Perspective effect in LEFT retrosplenial cortex connectivity

**Brain region**	**Side**	**MNI-coordinates (mm)**			**#voxels**	***T***	***Peak level***
		**X**	**Y**	**Z**			p_{FWE}
**Allocentric > egocentric**							
Middle Frontal Gyrus	L	-48	14	37	550	6.31	<0.001
Anterior Insula	R	33	26	-2	74	5.84	<0.001
Superior Parietal Lobule	L	-42	-49	52	353	6.67	<0.001
Calcarine Cortex	L	-9	-67	10	1106	6.93	<0.001
**Egocentric > allocentric**							
Anterior Cingulate Gyrus	L	-9	44	7	700	5.94	<0.001
Superior Frontal Gyrus	R	15	-10	67	56	5.06	0.018
Precentral Gyrus	L	-24	-10	70	1237	5.61	0.001
Postcentral Gyrus	R	48	-19	40	310	5.09	0.015
Central Operculum	L	-45	-4	7	78	5.92	<0.001
Fusiform Gyrus	L	-24	-52	-17	228	5.76	<0.001
Fusiform Gyrus	R	30	-43	-23	331	4.95	0.026

Supplementary Table 9: Perspective effect in RIGHT retrosplenial cortex connectivity

**Brain region**	**Side**	**MNI-coordinates (mm)**			**#voxels**	***T***	***Peak level***
		**X**	**Y**	**Z**			p_{FWE}
**Allocentric > egocentric**							
Precuneus	R	3	-70	34	763	6.20	<0.001
**Egocentric > allocentric**							
Supplementary Motor Cortex	L	0	-4	58	349	5.12	0.013
Central Operculum	L	-45	-1	4	57	5.06	0.017

Supplementary Table 10: Perspective effect in LEFT V1 connectivity

**Brain region**	**Side**	**MNI-coordinates (mm)**			**#voxels**	***T***	***Peak level***
		**X**	**Y**	**Z**			p_{FWE}
**Allocentric > egocentric**							
Middle Frontal Gyrus	L	-24	11	49	1046	5.70	0.001
Middle Frontal Gyrus	R	36	14	31	377	5.28	0.006
Supramarginal Gyrus	L	-45	-49	52	496	5.88	<0.001
Middle Temporal Gyrus	L	-54	-55	-8	153	5.08	0.016

Inferior Temporal Gyrus	R	54	-58	-14	45	4.87	0.046
**Egocentric > allocentric**							
Precentral Gyrus	L	-39	-22	55	282	5.05	0.019
Central Operculum	R	48	-4	4	69	5.15	0.011
Parietal Operculum	L	-45	-22	16	233	5.02	0.022

Supplementary Table 11: Perspective effect in RIGHT V1 connectivity

**Brain region**	**Side**	**MNI-coordinates (mm)**			**#voxels**	***T***	***Peak level***
		**X**	**Y**	**Z**			p_{FWE}
**Allocentric > egocentric**							
Middle Frontal Gyrus	R	30	14	55	83	5.11	0.014
Precentral Gyrus	L	-45	8	31	633	6.09	<0.001
Angular Gyrus	L	-30	-76	40	486	6.84	<0.001
Angular Gyrus	R	36	-73	40	216	6.29	<0.001
Cuneus	L	-9	-73	22	1366	6.98	<0.001
Middle Temporal Gyrus	L	-57	-58	-8	131	5.71	<0.001
**Egocentric > allocentric**							
Inferior Occipital Gyrus	L	-30	-91	-5	78	5.37	0.003
Occipital Pole	R	15	-97	-8	78	5.07	0.016

Supplementary Table 12: Strategy effect in LEFT retrosplenial cortex connectivity

**Brain region**	**Side**	**MNI-coordinates (mm)**			**#voxels**	***T***	***Peak level***
		**X**	**Y**	**Z**			p_{FWE}
**Euclidian > landmark**							
Occipital Fusiform Gyrus	R	30	-70	-8	374	5.19	0.009
Precuneus	L	-12	-61	28	453	4.91	0.038
**Landmark > Euclidian**							
Precentral Gyrus	L	-51	-7	46	59	7.34	<0.001
Supplementary Motor Cortex	L	-3	2	67	160	6.24	<0.001
Caudate	L	-15	5	10	314	5.76	<0.001
Putamen	R	18	8	1	86	5.14	0.012
Lingual Gyrus	L	-15	-73	-5	102	5.36	0.004

Supplementary Table 13: Strategy effect in RIGHT retrosplenial cortex connectivity

**Brain region**	**Side**	**MNI-coordinates (mm)**			**#voxels**	***T***	***Peak level***
		**X**	**Y**	**Z**			p_{FWE}
**Euclidian > landmark**							
Calcarine Cortex	R	12	-91	1	304	5.06	0.018
**Landmark > Euclidian**							

Precentral Gyrus	L	-48	-7	46	63	6.53	<0.001
Supplementary Motor Cortex	L	-3	2	67	144	5.78	<0.001
Caudate	L	-15	5	10	242	5.32	0.005
Putamen	R	18	8	1	115	5.31	0.005
Lingual Gyrus	L	-3	-85	-5	87	4.95	0.005

Supplementary Table 14: Strategy effect in LEFT V1 connectivity

**Brain region**	**Side**	**MNI-coordinates (mm)**			**#voxels**	***T***	***Peak level***
		**X**	**Y**	**Z**			p_{FWE}
**Euclidian > landmark**							
Putamen	L	-24	-1	7	148	6.00	<0.001
Inferior Occipital Gyrus	R	45	-76	1	468	5.12	0.013
Middle Occipital Gyrus	L	-33	-82	7	407	5.87	<0.001
Parietal Operculum	L	-48	-37	16	112	5.02	0.021
**Landmark > Euclidian**							
Inferior Frontal Gyrus	L	-36	14	25	256	5.52	0.002
Precuneus	L	-6	-52	49	136	5.56	0.001
Precuneus	L	-18	-64	22	77	5.39	0.003

Supplementary Table 15: Strategy effect in RIGHT V1 connectivity

**Brain region**	**Side**	**MNI-coordinates (mm)**			**#voxels**	***T***	***Peak level***
		**X**	**Y**	**Z**			p_{FWE}
**Euclidian > landmark**							
Supplementary Motor Cortex	L	-3	-1	64	134	6.12	<0.001
Superior Occipital Gyrus	R	21	-91	16	1083	8.36	<0.001
**Landmark > Euclidian**							
Middle Frontal Gyrus	R	33	59	10	41	4.91	0.038
Superior Frontal Gyrus	R	3	38	37	359	5.58	0.001
Anterior Insula	L	-30	20	-5	83	5.62	0.001
Lingual Gyrus	L	-18	-79	-14	275	6.02	<0.001
Angular Gyrus	R	42	-61	55	165	5.01	0.023

Supplementary Table 16: Sex differences in LEFT hippocampus connectivity

**Brain region**	**Side**	**MNI-coordinates (mm)**			**#voxels**	***T***	***Peak level***
		**X**	**Y**	**Z**			p_{FWE}
**Women > men**							
Superior Frontal Gyrus Medial	L	-3	59	-2	1594	8.34	<0.001
Frontal Operculum	L	-45	26	-2	122	5.45	0.002
Central Operculum	R	54	-13	10	133	5.19	0.009
Angular Gyrus	L	-42	-70	40	358	7.07	<0.001
Angular Gyrus	R	42	-67	43	125	5.15	0.011

Posterior Cingulate Gyrus	L	0	-46	16	502	7.10	<0.001
Middle Temporal Gyrus	R	66	-16	-17	85	5.38	0.003
Temporal Pole	R	33	5	-44	43	5.23	0.007
Hippocampus/Parahippocampus	L	-21	-19	-11	268	6.71	<0.001
Hippocampus/Parahippocampus	R	24	-19	-11	80	5.16	0.011
**Men > women**							
Middle Frontal Gyrus	R	45	47	19	222	6.56	<0.001
Middle Frontal Gyrus	R	33	8	64	272	6.22	<0.001
Supplementary Motor Cortex	R	3	8	52	93	5.21	0.008
Precentral Gyrus	L	-27	-7	46	133	5.57	0.001
Superior Parietal Lobule	L	-33	-46	64	469	5.84	<0.001
Supramarginal Gyrus	L	-48	-31	37	57	5.50	0.002
Supramarginal Gyrus	R	54	-31	43	93	6.06	<0.001
Inferior Temporal Gyrus	L	-39	-37	-17	62	5.23	0.007
Entorhinal Area	L	-18	-1	-32	104	5.94	<0.001
Lingual Gyrus	R	24	-43	-8	442	5.65	0.001
Subcallosal Area	R	3	17	-17	96	5.34	0.004

Supplementary Table 17: Sex differences in RIGHT hippocampus connectivity

**Brain region**	**Side**	**MNI-coordinates (mm)**			**#voxels**	***T***	***Peak level***
		**X**	**Y**	**Z**			p_{FWE}
**Women > men**							
Triangular Part of the Inferior Frontal Gyrus	L	-51	26	10	1831	7.51	<0.001
Basal Forebrain	R	27	5	-17	55	5.52	0.002
Angular Gyrus	L	-42	-67	37	268	6.25	<0.001
Posterior Cingulate Gyrus	L	0	-49	16	435	6.02	<0.001
Middle Temporal Gyrus	L	-60	-22	-23	356	5.87	<0.001
Middle Temporal Gyrus	R	54	-19	-17	65	5.33	0.004
Temporal Pole	L	-39	8	-41	84	6.11	<0.001
Hippocampus/Parahippocampus	L	-18	-22	-11	131	5.35	0.004
Hippocampus/Parahippocampus	R	24	-19	-11	142	7.60	<0.001
**Men > women**							
Superior Parietal Lobule	R	27	-52	52	165	5.81	<0.001
Superior Parietal Lobule	L	-21	-58	52	134	5.39	0.003
Medial Frontal Cortex	L	-6	23	-20	58	5.15	0.011

Supplementary Table 18: Sex differences in LEFT retrosplenial cortex connectivity

**Brain region**	**Side**	**MNI-coordinates (mm)**			**#voxels**	***T***	***Peak level***
		**X**	**Y**	**Z**			p_{FWE}
**Women > men**							
Superior Frontal Gyrus	L	-6	11	70	92	5.98	<0.001
Superior Frontal Gyrus	R	0	59	-2	247	6.25	<0.001

Left Middle Frontal	L	-24	35	37	52	5.47	0.002
Precentral Gyrus	L	-39	2	55	90	5.19	0.009
Middle Temporal Gyrus	R	63	-13	-11	54	5.16	0.011
Cuneus	R	3	-79	40	1419	9.63	<0.001
**Men > women**							
Anterior Orbital Gyrus	R	33	59	-14	80	5.83	<0.001
Superior Parietal Lobule	L	-18	-49	70	41	4.94	0.032
Occipital Pole	R	18	-91	13	41	5.15	0.011

Supplementary Table 19: Sex differences in RIGHT retrosplenial cortex connectivity

**Brain region**	**Side**	**MNI-coordinates (mm)**			**#voxels**	***T***	***Peak level***
		**X**	**Y**	**Z**			p_{FWE}
**Women > men**							
Triangular Part of the Inferior Frontal Gyrus	L	-57	26	4	124	5.03	0.020
Superior Frontal Gyrus	L	-9	11	67	118	5.57	0.001
Precentral Gyrus	L	-42	2	52	296	6.28	<0.001
Middle Temporal Gyrus	L	-66	-37	-11	644	6.84	<0.001
Angular Gyrus	R	57	-55	19	41	4.92	0.036
Cuneus	R	3	-79	40	939	9.48	<0.001
Lingual Gyrus	R	3	-61	7	78	10.74	<0.001
Inferior Occipital Gyrus	R	39	-91	-2	52	5.84	<0.001
**Men > women**							
Occipital Pole	R	15	-94	16	80	6.16	<0.001
Occipital Pole	L	-3	-100	-5	82	6.34	<0.001

Supplementary Table 20: Sex differences in LEFT caudate connectivity

**Brain region**	**Side**	**MNI-coordinates (mm)**			**#voxels**	***T***	***Peak level***
		**X**	**Y**	**Z**			p_{FWE}
**Women > men**							
Caudate	R	12	14	13	1112	9.21	<0.001
Planum Polare	L	-42	2	-11	864	6.17	<0.001
Planum Polare	R	45	2	-14	84	5.22	0.008
**Men > women**							
Superior Parietal Lobule	R	39	-52	61	48	5.91	<0.001

Supplementary Table 21: Sex differences in RIGHT caudate connectivity

**Brain region**	**Side**	**MNI-coordinates (mm)**			**#voxels**	***T***	***Peak level***
		**X**	**Y**	**Z**			p_{FWE}
**Women > men**							
Superior Temporal Gyrus	L	-57	-25	1	252	5.04	0.020
Middle Temporal Gyrus	L	-60	-58	7	103	5.58	0.001

Caudate	R	12	14	10	1031	9.56	<0.001
Planum Polare	R	45	5	-14	58	4.93	0.034
**Men > women**							
Precentral Gyrus	R	54	11	28	52	5.15	0.011

Supplementary Table 22: Sex differences in LEFT V1 connectivity

**Brain region**	**Side**	**MNI-coordinates (mm)**			**#voxels**	***T***	***Peak level***
		**X**	**Y**	**Z**			p_{FWE}
**Women > men**							
Occipital Fusiform Gyrus	L	-15	-94	-14	461	11.07	<0.001
**Men > women**							
Superior Frontal Gyrus (medial segment)	R	12	44	-5	718	5.45	0.002
Middle Frontal Gyrus	R	24	32	40	177	5.73	<0.001
Superior Occipital Gyrus	L	-18	-94	25	952	7.90	<0.001
Angular Gyrus	L	-36	-67	19	122	5.36	0.004
Inferior Occipital Gyrus	L	-45	-79	-14	97	6.28	<0.001
Inferior Occipital Gyrus	R	57	-64	-5	146	5.93	<0.001

Supplementary Table 23: Sex differences in RIGHT V1 connectivity

**Brain region**	**Side**	**MNI-coordinates (mm)**			**#voxels**	***T***	***Peak level***
		**X**	**Y**	**Z**			p_{FWE}
**Women > men**							
Lingual Gyrus	R	6	-94	-5	230	11.82	<0.001
**Men > women**							
Lingual Gyrus	R	9	-82	-2	711	8.92	<0.001
Middle Occipital Gyrus	L	-33	-94	10	289	6.37	<0.001
Angular Gyrus	L	-45	-70	22	94	4.93	<0.001
Middle Temporal Gyrus	R	57	-61	13	89	4.89	<0.001
