# Supplementary material for: Sex and strategy effects on brain activation during a 3D-navigation task
Source: Commun Biol. 2022 Mar 16;5:234. doi: 10.1038/s42003-022-03147-9 (PMC8927599; doi:10.1038/s42003-022-03147-9)
Supplement: Supplementary file 3 — Reporting Summary [file 42003_2022_3147_MOESM3_ESM.pdf]

nature research

Corresponding author(s): Isabel Noachtar & Belinda Pletzer

Last updated by author(s): 23/11/21

Reporting Summary

Nature Research wishes to improve the reproducibility of the work that we publish. This form provides structure for consistency and transparency in reporting. For further information on Nature Research policies, see our Editorial Policies and the Editorial Policy Checklist.

Statistics

For all statistical analyses, confirm that the following items are present in the figure legend, table legend, main text, or Methods section.

n/a	Confirmed	
☐	☒	The exact sample size (n) for each experimental group/condition, given as a discrete number and unit of measurement
☐	☒	A statement on whether measurements were taken from distinct samples or whether the same sample was measured repeatedly
☐	☒	The statistical test(s) used AND whether they are one- or two-sided<br>*Only common tests should be described solely by name; describe more complex techniques in the Methods section.*
☐	☒	A description of all covariates tested
☐	☒	A description of any assumptions or corrections, such as tests of normality and adjustment for multiple comparisons
☐	☒	A full description of the statistical parameters including central tendency (e.g. means) or other basic estimates (e.g. regression coefficient) AND variation (e.g. standard deviation) or associated estimates of uncertainty (e.g. confidence intervals)
☐	☒	For null hypothesis testing, the test statistic (e.g. F, t, r) with confidence intervals, effect sizes, degrees of freedom and P value noted<br>*Give P values as exact values whenever suitable.*
☒	☐	For Bayesian analysis, information on the choice of priors and Markov chain Monte Carlo settings
☐	☒	For hierarchical and complex designs, identification of the appropriate level for tests and full reporting of outcomes
☐	☒	Estimates of effect sizes (e.g. Cohen's d, Pearson's r), indicating how they were calculated

Our web collection on statistics for biologists contains articles on many of the points above.

Software and code

Policy information about availability of computer code

Data collection	Stimuli were created and presented using the Unreal Engine 4 Version 12. Neuroimaging data were acquired on a Siemens Magnetom Trio Tim 3 Tesla scanner.
Data analysis	Behavioral data and ROI-based activation were analyzed using the lme function of nlme package (Version 1.1-12) of statistics software R 3.3.2. & for the MRI-data we used Statistical Parametric Mapping package (SPM12, https://www.fil.ion.ucl.ac.uk/spm/software/spm12).

For manuscripts utilizing custom algorithms or software that are central to the research but not yet described in published literature, software must be made available to editors and reviewers. We strongly encourage code deposition in a community repository (e.g. GitHub). See the Nature Research guidelines for submitting code & software for further information.

Data

Policy information about availability of data

All manuscripts must include a data availability statement. This statement should provide the following information, where applicable:
- Accession codes, unique identifiers, or web links for publicly available datasets
- A list of figures that have associated raw data
- A description of any restrictions on data availability

Data and scripts for ROI-analyses are openly available at http://webapps.ccns.sbg.ac.at/OpenData/. MR-images for whole-brain analyses are available from the corresponding author upon reasonable request.

Field-specific reporting

Please select the one below that is the best fit for your research. If you are not sure, read the appropriate sections before making your selection.

☒ Life sciences ☐ Behavioural & social sciences ☐ Ecological, evolutionary & environmental sciences

For a reference copy of the document with all sections, see nature.com/documents/nr-reporting-summary-flat.pdf

Life sciences study design

All studies must disclose on these points even when the disclosure is negative.

Sample size	72 participants; power simulations for linear mixed effects models were run using the longpower package in R.
Data exclusions	No participant was excluded from the analyses.
Replication	N/A
Randomization	Participants were allocated to the experimental group by sex. Probands were tested three times and the versions of the navigation tasks were counterbalanced. In the analysis, we controlled for the session and IQ.
Blinding	Investigators were not blinded to group allocation during data collection and analysis.

Reporting for specific materials, systems and methods

We require information from authors about some types of materials, experimental systems and methods used in many studies. Here, indicate whether each material, system or method listed is relevant to your study. If you are not sure if a list item applies to your research, read the appropriate section before selecting a response.

Materials & experimental systems

n/a	Involved in the study	
☒	☐	Antibodies
☒	☐	Eukaryotic cell lines
☒	☐	Palaeontology and archaeology
☒	☐	Animals and other organisms
☐	☒	Human research participants
☒	☐	Clinical data
☒	☐	Dual use research of concern

Methods

n/a	Involved in the study	
☒	☐	ChIP-seq
☒	☐	Flow cytometry
☐	☒	MRI-based neuroimaging

Human research participants

Policy information about studies involving human research participants

Population characteristics	72 healthy participants: 36 men (mean age = 25.83 years, SD = 3.35 years) & 36 women (mean age = 26.39 years, SD = 4.35 years)
Recruitment	Participants were recruited via advertisement (broadcast emailing & posters) at the Faculty of Natural Science (University of Salzburg).
Ethics oversight	University of Salzburg’s ethics committee

Note that full information on the approval of the study protocol must also be provided in the manuscript.

Magnetic resonance imaging

Experimental design

Design type	Task-based, block design
Design specifications	This experiment included a 2x2 block design. The navigation task consisted of 20 items, each containing information about the perspective (either allocentric or egocentric reference) and about the strategy (either landmark-based or Euclidian reference).
Behavioral performance measures	Number of target reached during the navigation task

Acquisition

Imaging type(s)	Functional & structural
Field strength	3 Tesla
Sequence & imaging parameters	functional (using a T2*-weighted gradient echo planar (EPI) sequence sensitive to BOLD contrast):<br>TR = 2250 ms, TE = 30 ms, FOV 192 mm, matrix size 192x192, slice thickness = 3.0 mm, flip angle 70°, voxel size 3.0 x 3.0 x 3.0 mm, 36 transversal slices parallel to the AC-PC line<br>structural (using a T1-weighted sagittal 3D MPRAGE sequence):<br>TR = 2300 ms, TE = 2.91 ms, TI delay of 900 ms, FOV 256 mm, slice thickness = 1.00 mm, flip angle 9°, voxel size 1.0 x 1.0 x 1.0 mm, 160 sagittal slices
Area of acquisition	Whole brain

Diffusion MRI ☐ Used ☒ Not used

Preprocessing

Preprocessing software	AFNI for 3d-despiking (afni.nimh.nih.gov). SPM12 for realignment, co-registration, normalization and smoothing and first-level analysis, FIACH (Tierney et al., 2016) for physiological noise correction, CAT12 for segmentation. CONN-toolbox for connectivity analyses.
Normalization	Structural images were segmented and normalized using the computational anatomy toolbox (CAT12).
Normalization template	MNI152
Noise and artifact removal	3d-despiking, realignment, FIACH filtering, motion regressors and regressors of physiological noise during first-level analysis.
Volume censoring	N/A

Statistical modeling & inference

Model type and settings	A 2-stage mixed effects model was applied. By convolving the duration of the event with the canonical hemodynamic response function implemented in SPM12, we modeled one regressor per navigation category (allocentric-Euclidian, allocentric-landmark, egocentric-Euclidian, egocentric-landmark) in the subject-dependent fixed-effects first-level analysis.
Effect(s) tested	The subsequent analysis approach was two-fold. First, region of interest (ROI)-based analyses were performed by extracting principle eigenvariates as measures of BOLD-response from a one-sample T-test second-level design including all first-level contrast images. Eigenvalues were compared between sexes and conditions using linear mixed effects models. Second, differences in brain activation due to sex or condition were explored at the whole brain level. Contrast images (activation maps) were entered into a flexible factorial design modeling the factors sex, perspective and strategy as well as their interactions. Session was entered as covariate.

Specify type of analysis: ☐ Whole brain ☐ ROI-based ☒ Both

Anatomical location(s)	ROIs included the hippocampus, caudate, retrosplenial cortex and primary visual cortex and were defined based on Brodman areas in the Wake Forest University (WFU) Pickatlas toolbox.
Statistic type for inference (See Eklund et al. 2016)	We used an extent threshold of k = 40 voxels, an uncorrected primary threshold of $p < 0.001$ as well as a secondary peak-level FWE-corrected threshold of $p < 0.05$ (indicated as pFWE).
Correction	P-values for each ROI were FDR-corrected for multiple comparison.

Models & analysis

n/a	Involved in the study
☐	☒ Functional and/or effective connectivity
☒	☐ Graph analysis
☒	☐ Multivariate modeling or predictive analysis

Functional and/or effective connectivity	Connectivity analyses were performed using the CONN-toolbox. Seeds for ROI-to-voxel connectivity analyses were the left and right hippocampus, left and right retrosplenial cortex, left and right caudate, as well as the left and right primary visual cortex. Voxel-wise connectivity maps for each subject and session were entered into flexible factorial designs modeling the factors sex and condition as well as their interaction and session as a covariate.
